# Cardiac biomarkers as tools in the prediction and diagnosis of traumatic pericarditis and traumatic reticuloperitonitis in cattle and buffaloes

**DOI:** 10.1186/s12917-024-04174-w

**Published:** 2024-07-20

**Authors:** Heba A. Nasr, Nasr-Eldin M. Aref, Mahmoud R. Abd Ellah, Mohammed Ahmed Hamdy Abdelhakiem

**Affiliations:** 1 Department of Animal Medicine, Faculty of Veterinary Medicine, Assiut University, Assiut, 71526 Egypt; 2 Department of Surgery, Anesthesiology and Radiology, Faculty of Veterinary Medicine, Assiut University, Assiut, 71526 Egypt

**Keywords:** Cattle, Buffaloes, Traumatic pericarditis, Traumatic reticuleoperitonitis, Troponin

## Abstract

**Background:**

In the livestock industry, Foreign Body Syndrome is a devastating disease condition. Feeding management, lacking of food discrimination, and eating chopped food increase the risk of swallowing sharp foreign bodies in bovine species. In addition to the honeycomb cells shape of the reticulum, the contractions of the reticular wall, gravid uterine pressure, and parturition efforts, foreign bodies can penetrate the reticular wall, causing cascade of problems including traumatic reticulitis, traumatic reticuloperitonitis, and traumatic pericarditis. The present study was carried out to evaluate the diagnostic significance of cardiac troponin I rapid test cassette and other cardiac biomarkers including serum cardiac troponin I (cTn I), creatine kinase-myocardial band (CK-MB), lactate dehydrogenase (LDH), and aspartate aminotransferase enzyme (AST), in confirmed cases of traumatic pericarditis (TP) and/or traumatic reticuleoperitonitis (TRP) in cattle and buffaloes.

**Methods:**

A total number of 30 animals (22 cattle and 8 buffaloes) with different signs such as anorexia, jugular distension, brisket edema, and signs of pain (reluctance to move, arching back, and abduction of the forelimbs) were included in the present study. Based on case history, clinical signs, ferroscopic, pericardiocentesis, radiographic and ultrasonographic examinations, TP were confirmed in cattle (*n* = 10) and buffaloes (*n* = 8) while TRP were confirmed only in cattle (*n* = 12). Additionally, 20 clinically healthy animals (*n* = 10 cattle and 10 buffaloes) were used as a control group. Blood samples were collected for determination of blood level of Tn-I, and activity of CK-MB, LDH, and AST.

**Results:**

The obtained results revealed a highly significant increase in serum cTn I in diseased cattle with TP and TRP (*P* = 0.00), while buffaloes with TP showed no significant changes in serum cTn I (*P* = 0.111). Both diseased cattle and buffaloes showed increased serum activities of CK-MB, AST, and LDH enzyme. On the other hand, cardiac troponin I rapid test cassette failed to detect cTn I in diseased animals.

**Conclusion:**

The study concluded that the cardiac troponin I rapid test cassette did not have a diagnostic significance and could not be used as a point-of-care under field condition for diagnosis of TP and TRP in large ruminants. However, the serum troponin I level is helpful in diagnosis of TP and TRP in cattle. Although cardiac biomarkers have some diagnostic values in TP and TRP, the traditional diagnostic methods (clinical, radiography and ultrasonography examinations) are crucial for thorough evaluation of TP/TRP cases in bovine.

## Introduction

There is no doubt that cattle and buffaloes have economic importance in the world because they can utilize polysaccharides in plant cell walls to produce meat and milk for human consumption [1]. As a result of the indiscriminate behavior of ingestion, the problem of swallowing sharp or blunt foreign bodies remains one of the most important problems facing bovine producers. It accounts for a significant proportion of economic losses to livestock producers and affects the national economy [2]. Allied syndrome or hardware disease is one of the most important forestomach diseases in cattle and buffaloes [3, 4]. It is complicated diseased conditions among cattle and buffaloes that results in a wide range of serious disease conditions. Among of these conditions, traumatic reticuleoperitonitis (TRP) and traumatic pericarditis (TP) are common in cattle and buffaloes. It is a consequence of swallowing sharp foreign bodies that penetrate the reticulum, diaphragm, peritoneum, and pericardial sac [5, 6]. Traumatic pericarditis is a common sequela to the ingestion of sharp foreign bodies because of the proximity of reticulum to pericardium that facilitates the perforation process of a contaminated sharp foreign body and the initiation of inflammation [7–10]. An accumulation of serous or fibrinous inflammatory exudates in the pericardial sac can eventually lead to toxemia and heart failure [11–13]. The prognosis of bovine heart disease varies from guarded to poor, depending on its nature and severity [14]. The type, length, and direction of the penetrating foreign body determine the severity of these complications [15]. Therefore, early diagnosis of heart disease and the initiation of appropriate management, either by treatment or culling, can prevent or reduce economic losses.

Signalment, case history, and clinical examination are insufficient for the identification of foreign body diseases [16]. Radiography is widely employed for the diagnosis of abdominal and thoracic illnesses, particularly those caused by metallic foreign bodies [2, 17] and is an efficient tool for identifying and characterizing metal foreign bodies in and outside the reticulum. On the other hand, ultrasonography is the method of choice for detecting fibrinous deposits and abscesses that are hardly noticed by radiography [2]. Therefore, both tools are complementary [18, 19]; however their availability under field conditions is questionable.

Cardiac biomarkers such as cardiac troponin I (cTn I) and creatine kinase-myocardial band (CK-MB) may represent an alternative way for early diagnosis of TP in animals. Cardiac TnI is widely used in humans as an indicator of myocardial damage [20, 21]. Troponin is a protein complex of three subunits (T, I, and C) that are involved in the contractile process of skeletal and cardiac muscle. Both cardiac and skeletal muscle express troponin C; whereas troponin T and I are generally thought to be cardiac-specific [20, 21]. Additionally, creatine kinase-myocardial band (CK-MB) and lactate dehydrogenase type 1 (LDH1) are specific cardiac isoenzymes that increase in various cardiac disorders. However, in large animals, these biomarkers lack sensitivity and specificity compared to troponin [22].

Therefore, the present study aimed to evaluate the diagnostic significance of these cardiac biomarkers namely, cTnI, CK-MB, LDH and AST in confirmed cases of TP/TRP in both cattle and buffaloes with special reference to their prognostic value and how troponin mapping may function as a point-of-care in TP situations.

## Materials and methods

### Animals

This study was conducted on a total number of 50 animals (32 cows and 18 buffaloes) of both sexes and their ages ranged from 3 to7 years old. Twenty animals (*n* = 10 cows and 10 buffaloes) were clinically healthy (Control group) and belonging to Bani-Mor farm -Assiut Governorate and Veterinary Teaching Hospital (VTH), Assiut University- Egypt. Thirty animals (22 cows and 8 buffaloes, diseased group) were admitted to the VTH with one or more of the following histories: inappetence or anorexia, lethargy, brisket edema, jugular pulsation/distention, absence of rumination, sharp drop in milk yield, abduction of the forelimbs, and decrease in body weight.

### Clinical examinations

All animals were subjected to thorough clinical examinations, according to Jackson and Cockcroft [23]. A clinical chart was designed for taking a medical history and clinical examination of the admitted cases. It included body temperature, heart rate and rhythm, jugular pulsation or distension and presence of brisket edema, congested visible mucous membranes, diarrhea or constipation, and abdominal distention.

### Ferroscopic examination

The presented cases were inspected by guardian metal detector (Hand hold security metal detector, USA) for tentative diagnosis of swallowed metallic foreign body. A metal detector was applied over the ventral and ventrolateral aspect of caudal chest and cranial abdomen for detection of ferromagnetic foreign bodies (from the 3rd to the 9th intercostal spaces).

### Radiography

The presented cases were also subjected to examination by radiography to confirm the disorders (TRP/TP) using fixed radiographic apparatus (Philips, Super 80 CP, the Netherlands). A right or left lateral recumbent view was performed in animals. The radiographic factors were 60–80 KVs, 30–45 mAs, and FFD was 90 cm. The following criteria were considered radiographically: outline of the heart, size of the heart relative to the number of intercostal spaces, appearance and disappearance of caudal vena cava (CVC), the visualization of the triangular area between the CVC, caudal border of the heart and diaphragm, conspicuousness of the diaphragmatic line, and presence of abnormal structures under the line of diaphragm or in the cranial abdomen.

### Ultrasonography

Diseased animals were further subjected to ultrasonographic examination on both sides of the thorax from the 3rd to the 5th intercostal spaces in a standing position. The examined area was prepared through clipping and shaving of the hair, washing with warm water and soap, and then a coupling gel was applied. The forelimbs were pulled forward to facilitate the ultrasonographic examination using a 2.5 MHZ microconvex sector transducer (2.5-5 Microconvex transducer; MyLabTM one VET, Esoate, Italy). The right caudal long axis was mainly adopted for visualization of the four chambers of the heart. The cranial and caudal long axis on both sides were also used for evaluation of the right and left ventricular outflow with slight rotation of the transducer, according to Khalaphallah et al. [24]. The examined area was extended to the level of the 7th intercostal space for reticular evaluation. The examination was carried out according to what was reported in the previous studies [3, 4, 25, 26].

### Pericardiocentesis

Pericardiocentesis was performed according to Buczinski et al. and Imran et al. [27, 28]. Briefly, a sterile 14 gauge/ 5-inch stainless needle was used on the left parasternal region at 4–6 intercostal spaces, about 5–7 cm above the point of elbow under complete aseptic condition. Once the tip of the needle penetrated the outer pericardium into the pericardial space, the fluid came out or escaped from the hub of the needle.

### Blood samples

Two blood samples (whole blood and serum samples) were collected and prepared from all examined animals according to Coles [29]. Blood samples were collected from the jugular vein on a clean dry vacutainer tube containing heparin as an anticoagulant for screening of blood troponin by troponin card (ACRO BIOTECH, INC, U.S.A 4650 Arrow Highway, Suite D6 Montclair, CA 91,763 USA). Other blood samples were collected on clean and dry plain centrifuge tubes and allowed to flow freely and gently from the jugular vein over the inner surface of the tube. Samples were kept in the refrigerator at 4 °C for 30 min and then centrifuged at 3000 rpm for 15 min for the separation of serum. The collected sera were transferred into Eppendorf tubes, which were coded and kept at – 20 °C for determination of blood level of cTn I, and activity of CK-MB, LDH and AST. Samples with hemolysis were excluded from the study.

### Biochemical analysis of serum and plasma

#### Determination of troponin I (tn I) level

##### Cardiac troponin I rapid test (troponin card)

The cTn I rapid test cassette is a qualitative, membrane-based immunoassay for the detection of cardiac troponin I in serum, plasma, and whole blood. In this test procedure, capture reagent was immobilized in the test line region of the test. After the specimen was added to the specimen area of the cassette, it reacted with anti-cTnI antibody coated colloid gold particles in the test. This mix migrated chromatographically along the length of the test and reacted with the immobilized capture reagent. The test format detected cardiac troponin I in samples. If the sample included cardiac troponin, a colored line would emerge in the test line region to indicate a positive result. If the material lacked cardiac troponin, a colorful line would not emerge in this area, indicating a negative outcome. A colorful line emerged in the control region to act as a procedural check to signal that the appropriate volume of specimens had been introduced and membrane wicking had happened [30]. The minimal detection limit of the cardiac troponin I rapid test cassette is 0.5 ng/ml.

##### Bovine troponin I (Tn I) ELISA test

The test kit was obtained from Sinogeneclon Co., Ltd (China No.9 BoYuan Road, YuHang District 311,112, HangZhou, China) and was used for the quantitative measurement of bovine Tn-I in serum samples. The concentration of Tn-I in the samples was determined by comparing the optical density (OD) of the samples to the standard curve.

##### Determination of serum creatine kinase myocardial band (CK-MB) and lactate dehydrogenase (LDH) levels

Serum CK-MB and LDH were determined kinetically in the serum using spectrophotometer and by commercial kits supplied by spectrum (Spectrum Diagnostics, Cairo, Egypt) according to Young, [31].

##### Determination of aspartate aminotransferase enzyme (AST) activity

Serum activity of AST was determined kinetically in serum using spectrophotometer and by commercial kits supplied from Spectrum (Spectrum Diagnostics, Cairo, Egypt) according to Breuer, [32].

### Statistical analysis

In accordance with Borenstein et al., [33], the results were analyzed using the statistical package for the social sciences for Windows (SPSS, version 16.0). Data were presented as mean and standard error (M ± SE). One-way ANOVA was used to compare data from diseased cattle and buffaloes with those from the control group. The difference is considered significant when the *P*˂ 0.05, and highly significant when *P*˂ 0.001.

## Results

According to the findings of the case history, clinical signs, pericardiocentesis, radiography and ultrasonography examinations, TP was confirmed in 10 cases of cattle and 8 cases of buffaloes while TRP was confirmed only in cattle (*n* = 12).

### History and clinical findings

**Fig. 1:**
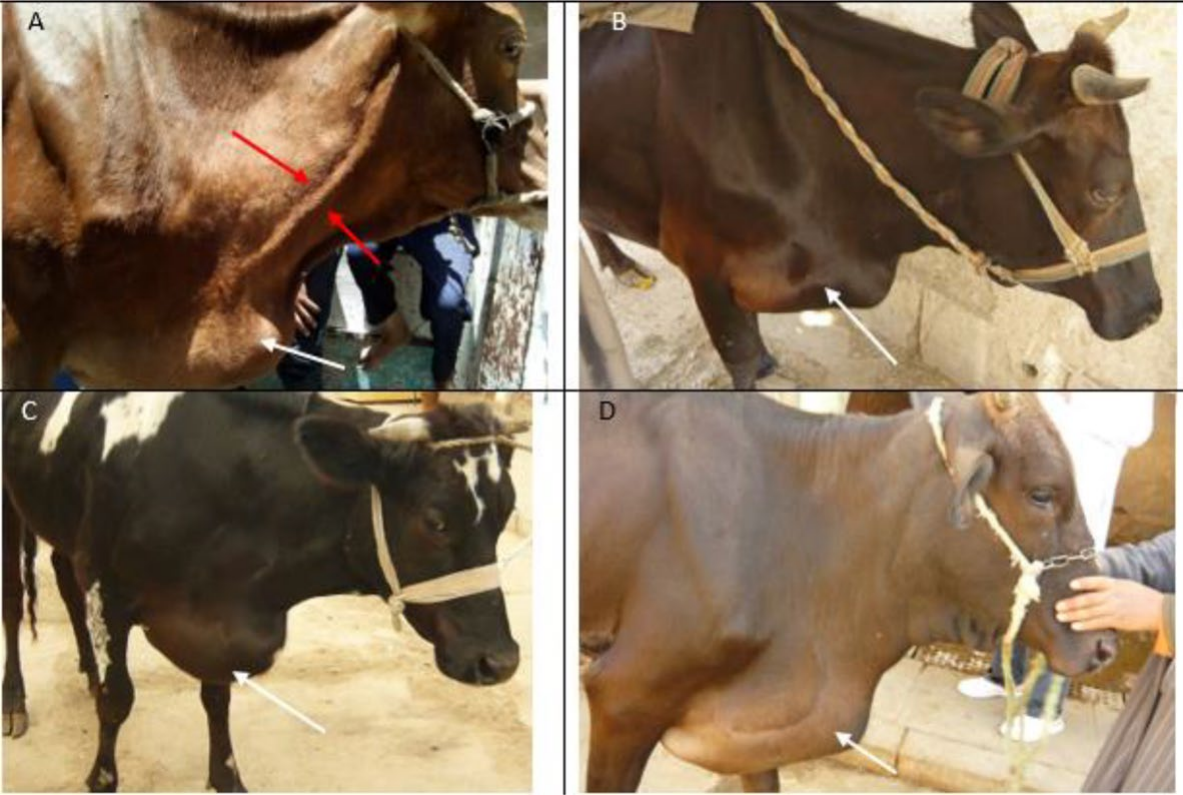
Images from **A** to **D** depicted cattle with traumatic pericarditis which is associated with an obvious jugular distension (**A**; between two red arrows), brisket edema (**A**-**D**; white arrows) and dullness of animals (**B**&**C**)

The main complaints and clinical signs of the presented cases were inappetence, anorexia or depraved appetite, decrease or absence of rumination, decreased ruminal movements or atony on auscultation, brisket edema, jugular pulsation (Fig. 1 (A-D), muffled heart sounds on auscultation, recumbency, congestion of mucous membranes, fever, abduction of the elbow, pain signs (grunting and tearing), dyspnea with tongue protrusion, reduction in milk yield and decrease of body weight. Dietary changes and improper feeding management were also recorded in case history. Ferroscopic examination revealed positivity in 19 cases (63.3%) (*n* = 15 cattle and 4 buffaloes) and false negative in 11 cases. Detailed clinical and ferroscopic findings were presented in Table 1.

**Table 1:** Clinical and ferroscopic findings of diseased cattle and buffaloes

	Cattle			Buffaloes	
	Control (*n* = 10)	TP (*n* = 10)	TRP (*n* = 12)	Control (*n* = 10)	TP (*n* = 8)
**Temperature**	37.5 ± 0.8	39.1 ± 1.45	38.3 ± 0.9	38 ± 0.91	38.9 ± 1.6
**Heart rate** **Beat/min**	58.4 ± 15.3	67.7 ± 8.57	63. ±11	64.8 ± 7.3	72.18 ± 8
**Ruminal motility**	3–4 cycle /2minmin	1 cycle /2min in 4 cases and stasis in 6 cases	1 cycle /2min in 8 cases and stasis in 4 cases	3 cycle /2minmin	1 cycle /2m in 7 cases and stasis in 1 casen
**Jugular distension**	-ve	+ve in all animals	-ve in all animals	-ve	+ve in all animals
**Mucous membranes**	Bright pink	Congested in 6 cases pale in 4n	Pale in 3 Normal in 3 Icteric in 1 Congested in 5	Bright pink	Congested in 6 cases pale in 2
**Defecation**	Normal	Normal in 4 Scanty in 5 Diarrheic in 1	Scanty in 3 No def. in 3 Normal in 5 Diarrheic in 1	Normal	Normal in 4 Scanty in 4
**Dehydration**	-ve	Mild to moderate	Mild to moderate	-ve	Mild to moderate
**Brisket edema**	-ve	+ve In all cases	-ve	-ve	+ve in all cases
**Ferroscopic findings**	-ve	5 positives 5 false negative	10 positives 2 false negative	-ve	4 positives 4 false negative

TP (traumatic pericarditis), TRP (Truamtic reticuleoperitonitis), -ve (negative), +ve (positive)

### Radiography findings

**Fig. 2:**
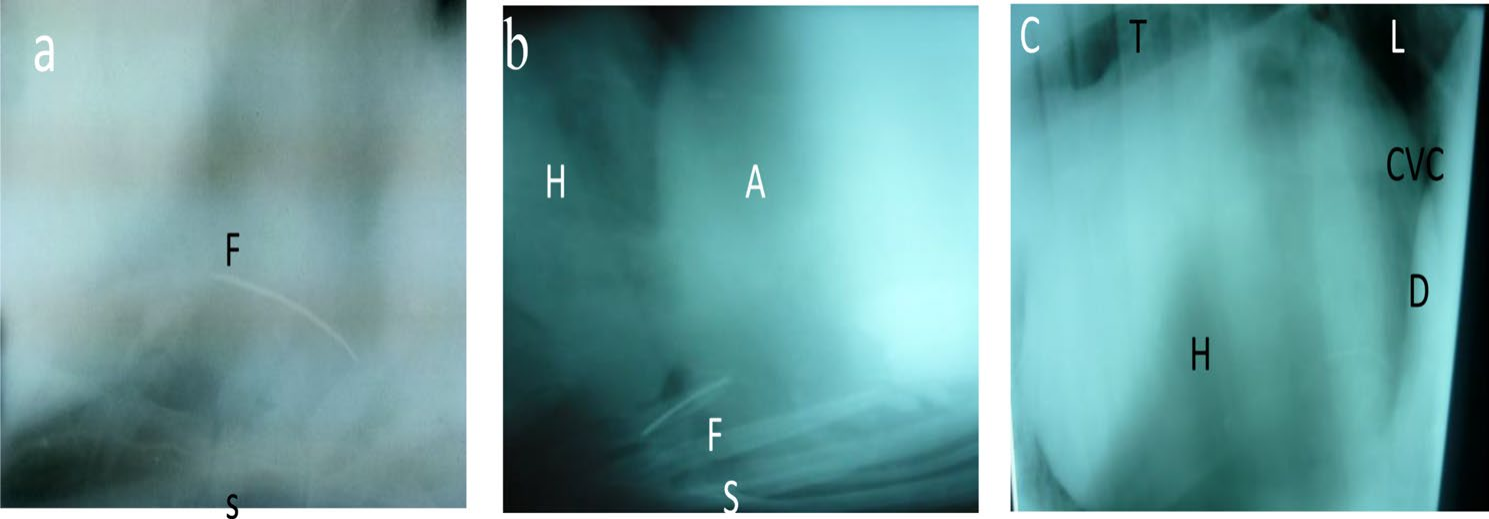
Lateral radiographic views of the thorax and cranial abdomen in cattle and buffaloes suffering Traumatic pericarditis displayed loss of details with visualization of foreign body (F) above the centrum of sternum (s) (**a**). The foreign body penetrated the diaphragm from abdomen (A) into the thorax and apex of heart (H) (**b**). The heart appeared enlarged than normal (**c**). (T= trachea, L= lung, CVC= caudal vena cava, D= diaphragm

**Fig. 3:**
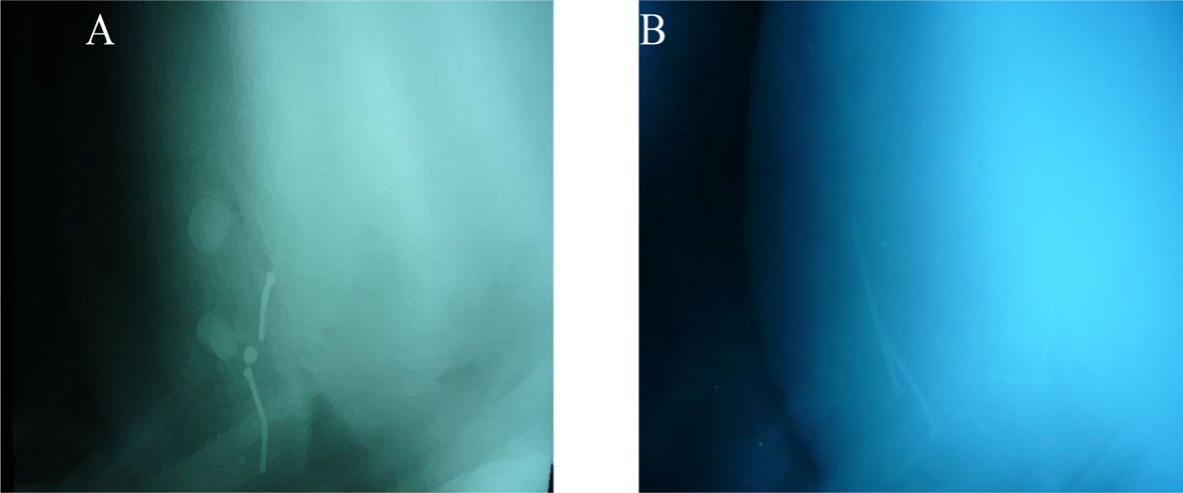
Lateral radiographs of a 4-years cow (**A**), and 5-years buffalo (**B**) showed radio-opaque foreign bodies (nails, rocks) within the cranial abdomen (reticulum) under the diaphragm

The nature and sequelae of the involved metallic foreign bodies and detailed description of X-ray findings in affected animals with TRP and TP were displayed in Figs. 2&3 and summarized in Table 2.

**Table 2:** Radiographic findings of diseased cattle and buffaloes

Inclusion criteria	TRP	TP in cattle	TP in buffaloes
**Nature of Foreign bodies**	2 case had needle 6 cases have nails 4 cases have wires	8 cases have needle 2 cases have nails	3 cases had needle 5 cases have nails
**Cardiac outline**	Clear and outstanding and easily to be demarcated	It differs than normal and could not be demarcated especially the caudal border which merge or blend with line of diaphragm causing what is called border effacement	It differs than normal and could not be demarcated especially the caudal border which merge or blend with line of diaphragm causing what is called border effacement
**Size of heart**	Normal (within from 2.5–3.5 intercostal spaces)	Large (2/10), and other cases due to its outline was not clear, the size could not be determined because of loss of details (8/10).	Large (5/8) and other cases due to its outline was not clear, the size could not be determined. There was a loss of details (3/8).
**Caudal vena cava (CVC)**	Clearly visualized	It was visualized in some animals (2/10) and could not be seen in others (8/10)	It was visualized in some animals but a very short part of it when emerging from the diaphragm into the heart (5/8) and could not be seen in others (3/8)
**Triangular area between CVC**,** Heart and Diaphragm**	It was clearly demarcated, and it decreased and increased according to the respiratory phase	It was determined in some animals (2/10) and it was radiopaque and merged cranially and caudally with heart and abdomen respectively in other animals (8/10)	It was determined in some animals (5/8) and it was radiopaque and merged cranially and caudally with heart and abdomen respectively in other animals (3/8)
**Visibility of Diaphragm’s line (DL)**	It was clear to be demarcated and sharp to separate between the abdomen and thorax	It was determined along its length except the most distal part (2/10), but it could not be demarcated in others (8/10)	It was determined along its length except the most distal part (5/8), but it could not be demarcated in others (3/8)
**Abnormal structure within thorax**	No	Needle (part or whole), nails (caudal part or tail)	Needle (part or whole), nails (caudal part or tail)

TP (traumatic pericarditis), TRP (traumatic reticuleoperitonitis)

### Ultrasonography findings

**Fig. 4:**
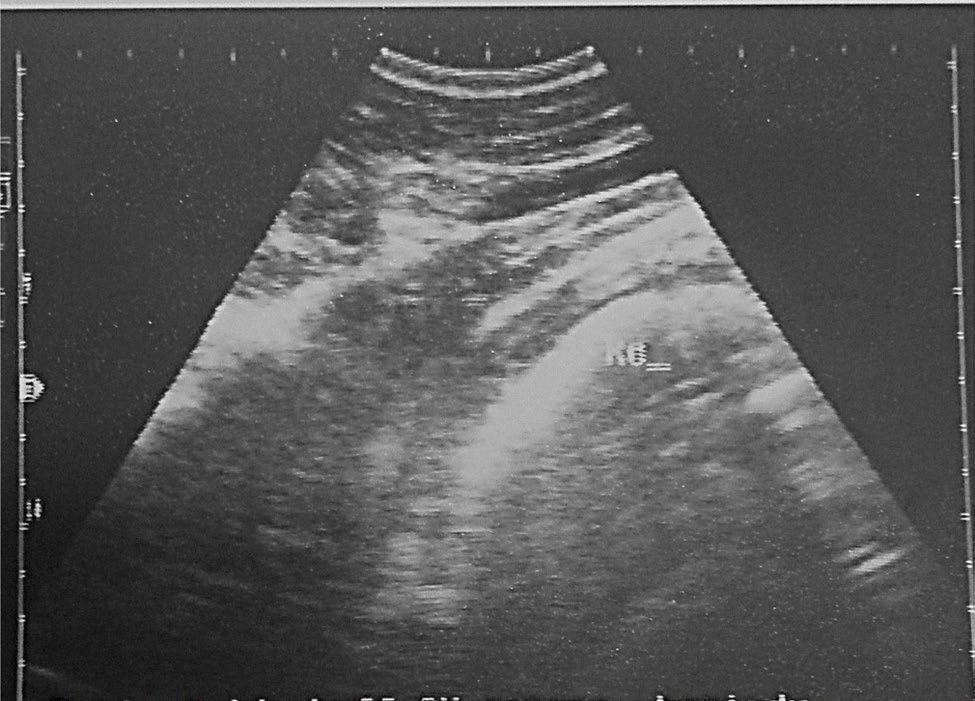
Ultrasonogram displayed echogenic material (Fibrinous inflammation) located ventral to the reticular wall (Re) which refers to TRP in a-6-year old non-pregnant animal

**Fig. 5:**
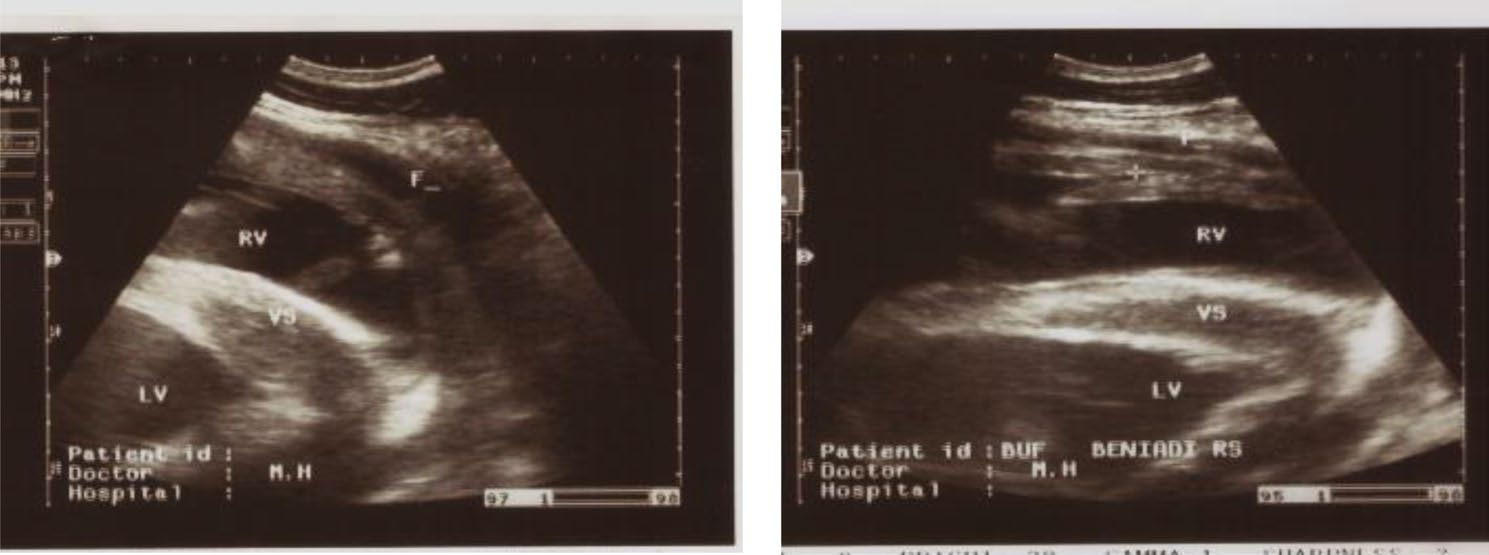
Ultrasonogram displayed the right (RV) and left ventricles (LV) of the heart, separated by interventricular septum (VS). The heart is surrounded by fluid (F) in a buffalo that suffered traumatic pericarditis

**Fig. 6:**
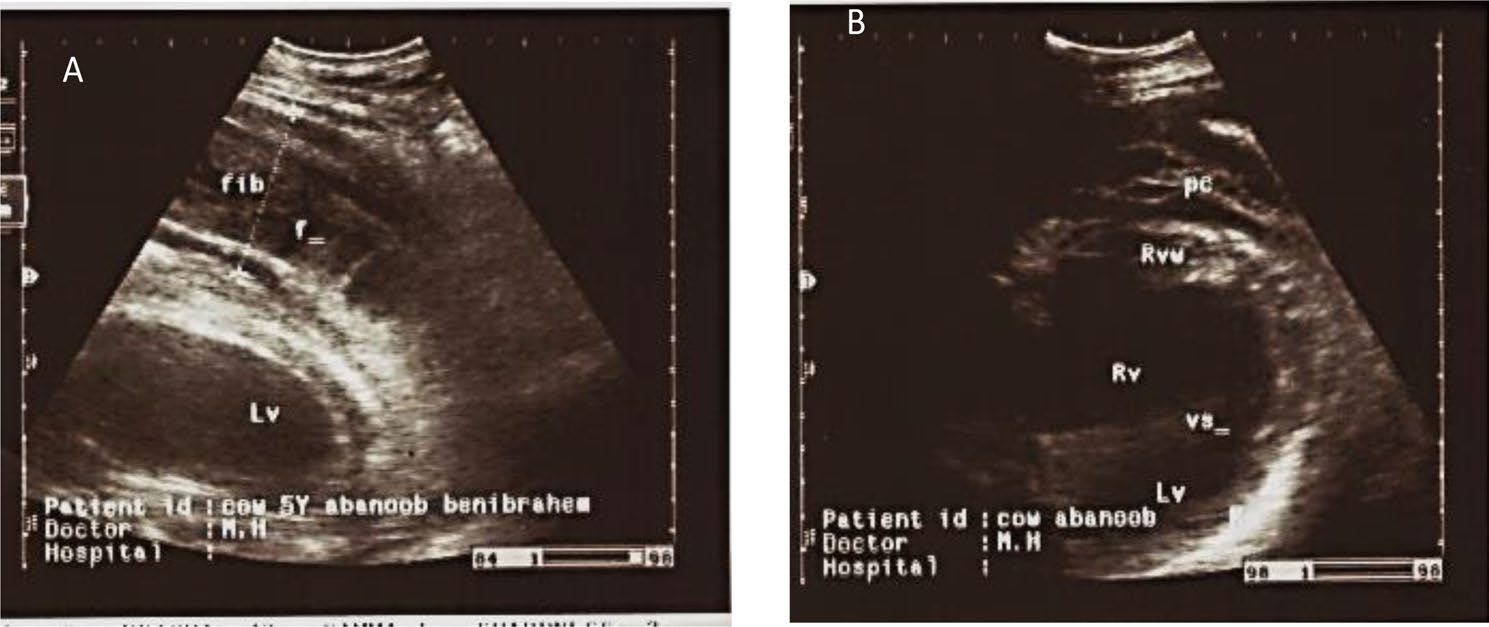
Ultrasonogram displayed the right (RV) and left ventricles (LV) of the heart, separated by interventricular septum (VS). The heart is surrounded by fluid (f) and fibrin (fib) in a cow that suffered traumatic pericarditis. **A**: Ultrasonogram from the left side; **B**: Ultrasonogram from the right side. The fluid (f) and fibrin fill the pericardial (pc) space. Rvw (right ventricular wall)

The main ultrasound findings in animals affected by traumatic pericarditis (TP) in cattle (4/10) were epicardial and pericardial effusions/ thickening around the heart, extended fibrin from the heart (2/10), increased echogenicity in the epicardium, in addition to hypoechoic content between pericardium and epicardium and increased echogenicity of the capsule. Additionally, in liver sonograph, there was severe congestion to the hepatic blood vessels, and change in the shape of caudal vena cava from triangular to large round. Lung was compressed and displaced medially and dorsally on sonogram because of the presence of pleural effusion. Fibrin strands and echogenic deposits were seen on the epicardium, while the pericardial anechoic fluid led to the reduction of the ventricular space because of compression. Hyperechogenic dots were visualized between the echogenic, thickened pericardium and epicardium suspended within the anechoic fluid (Figs. 4, 5 and 6). Ultrasound changes were also observed in cattle with TRP. The changes included the contour of the reticulum and presence of corrugation around its wall and presence of fibrinous adhesions between the reticulum and the dorsal sac of the rumen.

### Pericardiocentesis

**Fig. 7:**
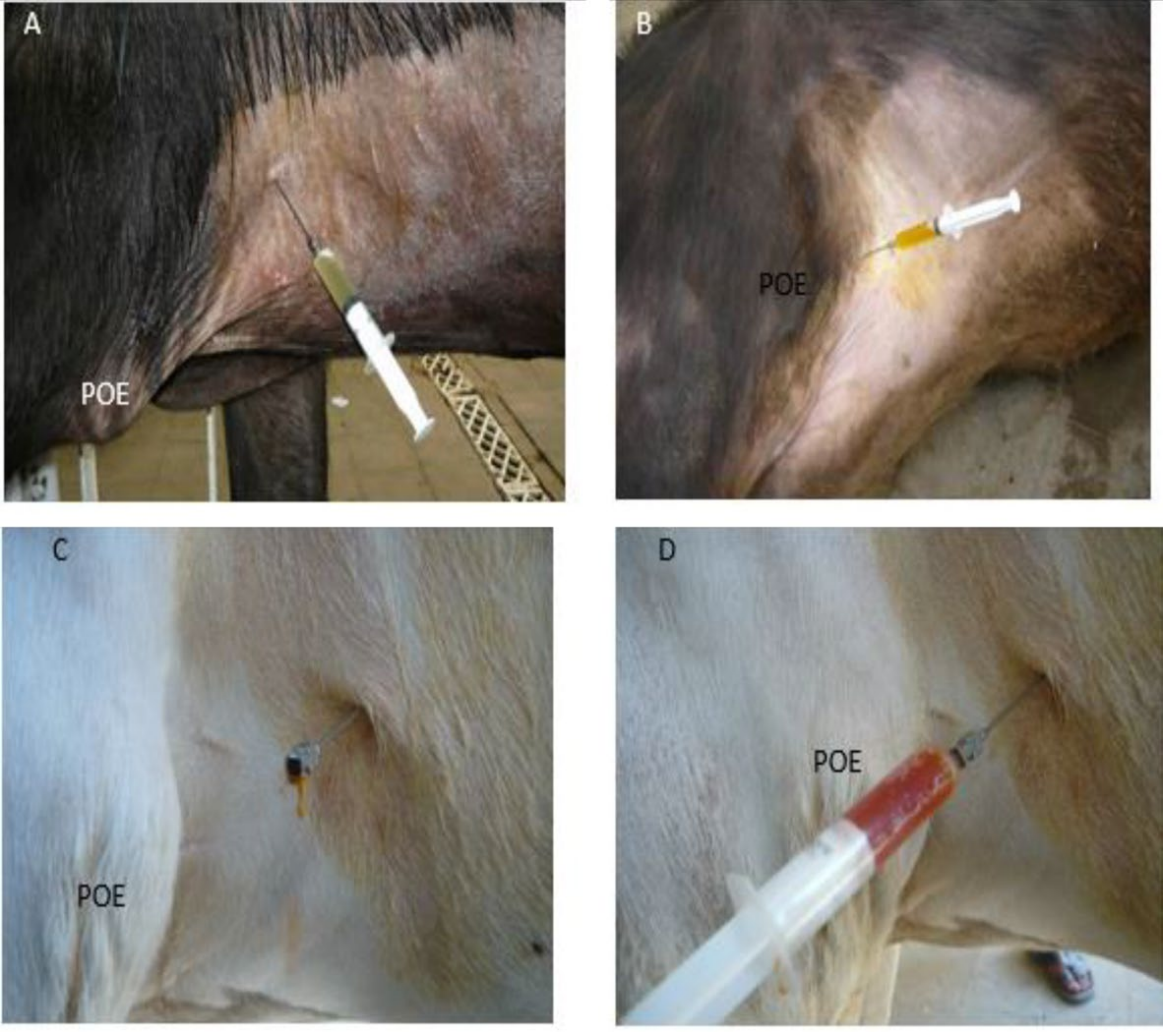
Different images (**A**-**D**) show the pericardiocentesis in cattle and buffaloes (The needle was inserted in the intercostal spaces (4-6), and about 5-7 cm above the point of elbow (POE) parasternal on the left side. The fluid comes out differ in color (Serosanguinous, yellowish to clear slight greenish color)

Eighteen cases showed the presence of fluids in the pericardial sac upon pericardiocentesis. There were copious amounts of fluid that varied in consistency, from clear watery fluid to turbid with flakes. The color was serosanguineous in some cases (6/18), light greenish in 6/18, and yellowish in 3/18. This is clear in Fig. 7A-D. The odor of the aspirated fluids was foul and offensive. The other remaining cases showed little amount of fluid on pericardiocentesis.

### Biochemical findings

Qualitative determination of cTn I using cardiac troponin I rapid test cassette (troponin card) did not show positive results in plasma, serum, or whole blood samples of diseased animals, and it did not have diagnostic significance in TRP and TP. On the other hand, quantitative evaluation of serum cTn I using bovine troponin I (Tn-I) ELISA test showed highly significant increase in diseased cattle with TP or TRP (*P* = 0.00), but showed no significant changes in diseased buffaloes with TP (*P* = 0.111). The present study revealed enzymatic changes in the serum of both diseased cattle and buffaloes, particularly AST and LDH. The obtained results displayed that the activities of serum CK-MB, LDH, and AST were significantly increasing in both diseased cattle with TP or TRP and diseased buffaloes with TP. Detailed biochemical findings were presented in Table 3.

**Table 3:** Serum troponin and enzymes in control and diseased cattle and buffaloes

	Cattle				Buffaloes		
	Control	TP	TRP	*P* value	Control	TP	*P* Value
TcI (ng/ml)	0.13 ± 0.03^c^	0.52 ± 0.10^a^	0.28 ± 0.03^b^	0.000	0.24 ± 0.03	0.31 ± 0.02	0.111
LDH (U/l)	305.40 ± 17.81^b^	528.60 ± 31.10^a^	459.29 ± 55.05^a^	0.003	322.21 ± 23.14	577.44 ± 29.27*	0.000
AST (U/l)	28.09 ± 4.39^b^	92.36 ± 4.12^a^	91.45 ± 6.21^a^	0.000	38.36 ± 5.54	98.27 ± 6.67*	0.000
CK-MB (U/l)	163.02 ± 12.63^b^	361.22 ± 17.84^a^	282.52 ± 29.42c	0.000	204.20 ± 19.80	348.14 ± 30.00*	0.001

Data are expressed as Mean ± SE. In each row, value followed by different superscript letter is significant (*P* < 0.05). Each animal species was compared separately

TP: Traumatic pericarditis; TRP: Traumatic reticuloperitonitis

## Discussion

Animals with TP in the present study showed signs of pain, jugular pulsation, brisket edema, abduction of the elbow and difficulty in movement, decreased inappetence and production of milk with loss of body weight, congestion of mucous membrane, fever, tachycardia, and muffled heart sound. These signs agreed with those reported before [2, 5, 8, 11, 14, 34–36].

The most characteristic signs of the animals suffering TP in this study were tachycardia, brisket edema, jugular distension, and muffled sounds of the heart. These signs depend primarily on the degree of compression of the heart by pericardial effusion. The heart sounds are muffled because of pericardial effusion and fibrinous changes in the pericardial sac [36, 37]. Additionally, edema of the brisket and ventral abdomen and various degrees of distension of the jugular veins depend on the degree of cardiac tamponade [36, 38, 39].

Animals with TRP in the present study showed inappetence, decreased or stopped milk production, increased body temperature, tachycardia, jugular pulsation in some animals, congestion of mucous membrane, tucked up appearance, and pain sensation on palpation. The pattern of animal movement varied between normal, reluctant to move and recumbency. Decrease ruminal motility or ruminal atony, and scanty feces were also recorded. These signs were consistent with those reported previously [2, 4, 40–45]. An elevated rectal temperature is indicative of a systemic reaction, an increase in the respiratory rate indicates respiratory distress associated with toxemia and septicemia caused by foreign body penetration. Ruminal movements were markedly depressed, indicating severe hypomotility of the rumen. Tachycardia and polypnea are occasionally associated with some contributing factors, such as increase in body temperature, decrease in feed intake and the potential development of dehydration, and acid base imbalance.

The ferroscopic examination showed that 63.3% of cases were true positive for existence of metal objects and 36.7% were false negative in confirmed cases of TRP/TP in cattle and buffaloes raising the issue of lack of sensitivity of guardian metal detector. These findings were reported before [36]. On the contrary, radiography was best suited for visualizing metallic foreign bodies in and outside the reticulum and obtaining accurate information about their nature and position [2, 46]. In this study, the radiographic examination played a great role in the diagnosis and differentiation between several ruminal disorders and was used to confirm the results of the ferroscopic examination.

Radiographic examination of animals with TP showed that the cardiac outline is different from normal and could not be demarcated. The size of the heart is large, caudal vena cava was visualized in some cases but not be seen in others and the presence of radiopaque abnormal structures such as needles, and wires in some cases showed poor thoracic details. These results were similar to those reported before in different studies [5, 7, 11, 27, 35]. The non-visualization of the object that may occur due to inflammatory reactions and the presence of fibrinous exudates in the pericardial sac; does not exclude the suspicion of the TP [35, 47].

Radiographic examination of cattle with TRP showed that the diaphragm and heart were clear and easily identified. The heart was within normal size and shape. The caudal vena cava was clearly visualized and traced between the heart and diaphragm. However, there were abnormal structures within the cranial abdomen such as nails, wires, and needles. Similar findings were reported in previous studies [40, 43, 48–50].

The main ultrasound findings of TP in the present study were pericardial effusions and thickening around the heart, extended fibrin from the heart, increased echogenicity in the epicardium, in addition to hypoechoic content between the pericardium and epicardium, and increased echogenicity of the capsule. Additionally, there was severe congestion in the hepatic blood vessels, and change in the shape of caudal vena cava from its triangular to large round shape. The lung was compressed and displaced medially and dorsally by the pleural effusion. These results are in accordance with those recorded previously [22, 37, 51–55]. The presence of adhesions and fibrin stuck to the epicardium indicates the chronicity of the inflammatory process resulting from foreign body penetration, whereas the hemorrhagic injury observed can be attributed to severe septicemia, endotoxemia, and anoxia [56]. Cardiac tamponade is a life-threatening hemodynamic condition resulting from pericardial effusions. Increasing intra-pericardial pressure because of pericardial effusion is sufficiently to compress and restrict cardiac chamber filling, constrain cardiac output, and induce backward failure. Reduced right ventricular diastolic filling increases the venous hydrostatic pressure, which leads to hepatic enlargement, congestion, as well as distension of the jugular veins and the caudal vena cava. Moreover, the surface of the pericardium was rugged, and echogenic pericardial projections were imaged throughout, which were indicative of chronic inflammatory changes.

Cattle with TRP showed sonographic abnormalities, including changes in the contour of the reticulum, corrugations around its wall, and fibrinous adhesions between the reticulum and the dorsal sac of the rumen. These findings are in agreement with those stated in previous studies [3, 4, 40, 41, 57–59]. In the presence of a perforating foreign body, peritonitis may develop, and adhesions may progress between the reticulum and the surrounding tissues (peritoneum, spleen, and abomasum) leading to reduction in reticular motility [60]. Moreover, the reduction of reticular motility or absence was a characteristic ultrasonographic feature of TRP [61]. The corrugation of the reticular wall is due to the accumulation of fibrinous deposits interspersed with fluid pockets on the reticular serosa.

Although pericardiocentesis is an invasive technique, it might be helpful to confirm the presence of pericardial effusion under field conditions. In the present study, pericardiocentesis displayed different colored fluids (yellowish, light greenish, and serosanguinous). Similar results were reported in previous studies [27, 28].

Cardiac biomarkers were evaluated in the present study for potential diagnosis of TRP/TP in cattle and buffaloes under field conditions. The cardiac troponin I (cTn I) is considered the gold standard test for diagnosis of myocardial injury in humans and animals [62]. However, the results of cardiac troponin I rapid testing were not diagnostically significant, probably because they were below the detection limit. These findings were partially in disagreement with those previously reported [63]. The authors found 11 positive cases and 9 negative cases of TRP in cattle. Moreover, the authors [63] added that these kits are not suitable for testing cTn I in bovines because they are insensitive to levels below 0.5 ng/ml [64]. Additionally, the authors [63] stated that the human based point-of-care lateral flow immunochromatographic test kits used to determine the concentration of cTn I were not satisfactory for bovine usage.

In the current study, serum cTn I level was also assessed by using Bovine troponin I (Tn-I) ELISA test. The obtained results showed that cattle with TP/TRP had significant increases in serum cTn I levels. These findings are consistent with those reported in previous studies [8, 44, 65–67]. However, no significant changes in serum cTn I levels were observed in buffaloes with TP. This finding could be attributed to the disease course and the time elapsed from the onset of the disease to case presentation. Where, most buffalos were admitted to our clinic after a long time from the onset of the clinical signs and after several trials of treatment in the field. It was reported that the high cTn I value is an indicator of myocardial damage and heart failure in cattle at an early stage of the disease, since its release into the circulation occurs within eight hours in response to any myocardial microinjury, and remains elevated for up to two weeks [62, 67]. Moreover, it was stated that cTn I value is higher in acute cases [65].

Cattle with TRP in the present study showed a significant increase in serum troponin I level. This result is in consistent with the outcomes of the previous studies [40, 63]. It was reported that the circulating cardiac troponin I (cTn I) and cardiac troponin T (cTn T) can be used to determine the myocardial cell damage in cattle with TRP. The potential relationship between the acute myocardial cell damage caused by TRP and circulating cTn has not been studied extensively [38]. However, the authors thought that the decrease of feed intake in animals with TRP for a long time may lead to a degree of dehydration and hypovolemia. This may result in a decrease of the venous return which in turn causes the compensatory increase of the heart rate. Eventually, it may affect the cardiac muscles (fatigue), and heart failure may develop if the problem sustained without medical or surgical intervention. The present study displayed that the activities of serum CK-MB, LDH, and AST were significant increased in cattle with TP and TRP. These results are in agreement with the results that were published before [5, 11, 35, 51, 62, 67]. The enzymatic changes observed in the serum of both cattle and buffaloes, particularly of the AST and LDH enzymes; might suggest liver impairment and hepatic congestion in animals with foreign body syndrome with or without cardiac involvement.

The results of the present work exhibited significantly increases in CK-MB in cattle and buffaloes suffering from TP and TRP. CK-MB is an isoenzyme of CK, which is more specific to the cardiac muscle. It has a higher specificity for myocardial cell injury [62, 68]. In contrast, it was stated that this enzyme may have a low correlation with cardiac changes because CK-MB can rise in other cases of muscle injury [69].

Diseased buffaloes with TP showed a significant increase in serum CK-MB, LDH, and AST activities. These results agree with those reported before [5, 8, 34, 44, 70]. Moreover, it was suggested that the elevations in serum concentrations of liver enzymes generally indicated chronic lesions associated with right heart failure with secondary hepatic congestion [37].

## Conclusion

The findings of the present study indicate that the rapid test cassette for cardiac troponin I cannot be relied upon for diagnosis of cardiac diseases in cattle and buffaloes. However, the level of serum troponin I increased in cattle affected by traumatic pericarditis and traumatic reticuloperitonitis, but it remains unaffected in buffaloes with traumatic pericarditis. While cardiac biomarkers have some diagnostic value in cases of TP and TRP, traditional diagnostic methods such as clinical, radiography, and ultrasonography examinations are essential for thoroughly evaluating TP/TRP cases in bovine.

## Data Availability

No datasets were generated or analysed during the current study.

## Abbreviations

TRP: Traumatic Reticuleoperitonitis
TP: Traumatic Pericarditis
cTn-I: Cardiac Troponin I
CK-MB: Creatine Kinase-Myocardial Band
LDH: Lactate Dehydrogenase
AST: Aspartate Aminotransferase Enzyme
